# TCR-Based Antigen-Specific Therapy for Type 1 Diabetes Mellitus: From Editing Autoreactive Clones to Tolerance Induction

**DOI:** 10.3390/ijms262311563

**Published:** 2025-11-28

**Authors:** Marina Fisher, Julia Philippova, Vasily Kurilin, Sergey Sennikov

**Affiliations:** Laboratory of Molecular Immunology, Federal State Budgetary Scientific Institution Research Institute of Fundamental and Clinical Immunology, 630099 Novosibirsk, Russia; msolshanova@gmail.com (M.F.); airyuka@mail.ru (J.P.); vkurilin@niikim.ru (V.K.)

**Keywords:** T-regulatory cells, T cell receptor, autoimmune diseases, immunological tolerance, TCR-Treg, antigen-specific therapy

## Abstract

Type 1 diabetes mellitus (T1DM) is an autoimmune disease caused by the destruction of insulin-producing pancreatic β-cells by autoreactive T cells. Current treatments, including insulin replacement therapy and various immunotherapies, often modulate but fail to permanently halt the underlying autoimmune process or restore β-cell function. In this review, we examine T cell receptor (TCR)-based treatment strategies for T1DM. We focus on two main approaches: selective elimination of pathogenic autoreactive T cell clones and induction of immune tolerance using TCR-modified regulatory T cells (TCR-Tregs). We describe key islet autoantigens, including post-translationally modified neoantigens such as fusion insulin peptides, which are crucial for identifying pathogenic TCRs. Next, we will review methodologies for TCR detection and TCR-Treg generation, highlighting their mechanisms of action and impact on various immune cells, including dendritic cells, B cells, and macrophages. We will also examine the potential of CD8^+^T cell regulatory cells (CD8^+^Tregs). Finally, we will discuss the future of TCR-based therapy, emphasizing the need to optimize TCR affinity, ensure Tregs’ stability, and develop combination therapies. TCR-based therapy represents a revolutionary approach to restoring immune tolerance in T1DM, providing high specificity and reducing the risk of systemic immuno-suppression compared to traditional treatments.

## 1. Introduction

Type 1 diabetes mellitus (T1DM) is a chronic autoimmune disease characterized by the targeted destruction of insulin-producing beta cells within the islets of Langerhans. T1DM affects approximately 9.5 million people worldwide in 2025, with an annual incidence of ~513,000 new cases (164,000 in ages 0–14 and 58,000 in 15–19 years), per the International Diabetes Federation (IDF) Atlas 11th Edition [1]. Incidence varies regionally, highest in Northern Europe (e.g., Finland: ~60/100,000 children/year) and rising in low-income countries by 2.4% annually. Mortality remains elevated, with standardized mortality ratios 3–18 times higher than the general population; remaining life expectancy for a 10-year-old diagnosed in 2025 ranges from 6 to 66 years across countries, largely due to cardiovascular and renal complications [1,2]. This pathological process is primarily driven by autoreactive T and B cells, which recognize and target specific islet autoantigens [3]. Upon activation by antigen-presenting cells (APCs), these autoreactive T cells orchestrate the progressive damage to pancreatic islets, leading to a sustained loss of beta cell function [4]. Specifically, CD8^+^cytotoxic T cells directly mediate beta cell lysis, while CD4^+^T helper cells contribute to beta cell impairment through cytokine secretion and cooperative interactions with CD8^+^T cells and B cells [5]. A hallmark of T1DM is the elevated frequency of autoreactive CD8^+^T cells coupled with a diminished capacity of immunoregulatory mechanisms within the immune system [6].

Current management of T1DM remains primarily symptomatic and relies on lifelong exogenous insulin replacement delivered via multiple daily injections or continuous sub-cutaneous insulin infusion systems, frequently combined with real-time continuous glucose monitoring (CGM) and hybrid closed-loop automated insulin delivery (AID) systems such as the Medtronic MiniMed 780G and Tandem t:slim X2 with Control-IQ and Dexcom G7 integration [7,8]. Adjunctive therapies include pramlintide for postprandial glucose control and off-label use of SGLT2 inhibitors (e.g., dapagliflozin) for cardiovascular and renal protection despite the increased risk of diabetic ketoacidosis [7]. The only approved disease-modifying therapy is the anti-CD3 monoclonal antibody teplizumab (Tzield), which delays progression from Stage 2 to clinical Stage 3 T1D by a median of 2–3 years in at-risk individuals [9,10,11]. Regenerative approaches have advanced with FDA approval in 2023 of allogeneic pancreatic islet transplantation (Lantidra/donislecel) for adults with severe hypoglycemia unawareness, while stem cell-derived islet replacement therapies (e.g., zimislecel, Vertex Pharmaceuticals) are in Phase 3 trials in 2025, achieving insulin independence in ≥80% of recipients at 1 year in pivotal studies [12,13]. Despite these advances, no curative treatment exists, and all current strategies carry risks of hypoglycemia, ketoacidosis, and long-term complications [7].

Antigen-specific cell therapy aims to selectively suppress or eliminate clones of pathogenic immune cells without inhibiting the overall immune response. We focus on antigen-specific Treg cells modified with TCRs offer the advantage of precisely editing autoreactive clones and inducing immune tolerance, potentially improving efficacy and safety compared with previously used strategies.

**Figure 1:**
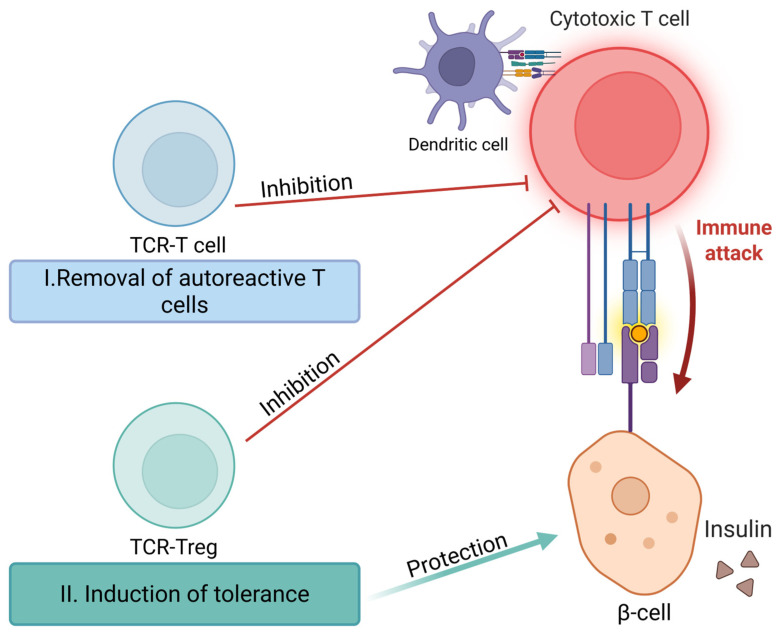
TCR- based antigen-specific therapeutic strategies for type 1 diabetes mellitus. Strategy 1: Selective elimination of autoreactive T cells via TCR-specific targeting. Strategy 2: Induction of immune tolerance employing TCR-Tregs.

TCR-based antigen-specific therapeutic strategies for T1DM can be divided into two directions (Figure 1): the selective removal of autoreactive T cells and the induction of immune tolerance through the application of TCR-Tregs.

This review will examine the principal autoantigens implicated in T1DM, discuss established and emerging methods for identifying pathogenic TCRs, and describe antigen-specific TCR-mediated strategies for T1DM treatment. Furthermore, we will elucidate the impact of these therapeutic approaches on other components of the immune system.

## 2. Key Autoantigens in T1DM and Pathogenic TCR Detection

For the development of antigen-specific therapeutic methods, such as TCR-mediated therapy, it is necessary to determine the specific reactivity of T cells to beta-cell antigen epitopes, followed by the isolation of pathogenic TCRs. Crucially, careful selection of TCRs with high specificity to the pathogenic antigen is required. The autoimmune response in T1DM is characterized by the progressive destruction of insulin-producing beta cells of the pancreas, mediated by T lymphocytes specific to several key islet autoantigens [14] (Table 1).

Insulin (INS) is the primary and earliest target of autoimmunity in T1DM. The immunodominant CD4^+^T cell epitope within the insulin B-chain is InsB: 9–23 (SHLVEALYLVCGERG), presented by HLA-DQ8 and HLA-DQ2 risk alleles [15]. Post-translational deamidation of this peptide markedly increases its binding affinity and immunogenicity [16]. For CD8^+^T cells, the glucose-regulated preproinsulin epitope PPI15–24 (ALWGPDPAAA), presented by HLA-A*02:01, is a major target. Skowera et al. demonstrated that CD8^+^ T cells from T1DM patients efficiently kill β-cells via recognition of this epitope using MHC class I tetramers [17]. This study, performed on a relatively small cohort (n = 16 patients), remains foundational and has been independently confirmed in larger cohorts using tetramer and functional assays; however, the exact contribution of this single epitope to overall β-cell destruction is still debated.

Glutamate decarboxylase 65 (GAD65) is a major autoantigen recognized predominantly by CD4^+^T cells. The most immunogenic region spans residues 555–567 (NMYAMMIARFKMFPEVKEKGMAALPRL) presented by HLA-DR4 [18]. Yang et al. showed that T cells from T1DM patients preferentially recognize a low-affinity register of InsB: 11–23, but for GAD65, the response is characteristically Th1-biased with high IFN-γ production. Although anti-GAD65 autoantibodies appear early and are stable diagnostic markers [19], their direct pathogenic role is limited; T cell reactivity to GAD65 epitopes correlates better with residual β-cell function preservation, making GAD65 an attractive target for antigen-specific tolerance induction.

Islet antigen-2 (IA-2, PTPRN) is a transmembrane protein of secretory granules. The dominant CD4^+^ T cell epitopes map to the C-terminal cytoplasmic domain, with the best-characterized being IA-2 752–775 and overlapping regions recognized by both autoantibodies and T cells [20]. Anti-IA-2 autoantibodies typically emerge later in the prediabetic phase and are associated with faster progression to clinical disease [21]. The overlap between B- and T cell epitopes, demonstrated by phage-display and molecular modeling [20], supports the concept of linked epitope recognition and makes IA-2 useful for combined humoral/cellular monitoring.

Zinc transporter 8 (ZnT8, SLC30A8) is the most recently identified major autoantigen. T- and B-cell epitopes cluster in the C-terminal domain (aa 268–369). The single nucleotide polymorphism rs13266634 (R325W) dramatically alters epitope immunogenicity: the 325R variant generates the dominant epitope ZnT8 318–331 (VAANIVLTVVAEFLR), while 325W reduces recognition [22]. Wenzlau et al. first described ZnT8 autoantibodies in 60–80% of new-onset T1DM patients, a finding robustly replicated worldwide. ZnT8 autoantibodies add significant predictive value when combined with INS, GAD65, and IA-2 antibodies.

**Table 1:** Classical Autoantigens in T1DM.

Autoantigen	Representative Key Epitope(s)	HLA Restriction	Role in Pathogenesis/Predictive Value	References and Comments
Insulin (INS)	InsB:9–23 (SHLVEALYLVCGERG); PPI15–24 (ALWGPDPAAA)	DQ8/DQ2; A*02:01	Earliest and primary target; post-translational modifications critical	[16,17]; Skowera et al. seminal but small cohort; findings widely reproduced
GAD65	GAD555–567	DR4	Strong Th1 CD4^+^response; autoantibodies stable marker	[18,19]; consistent T cell data across cohorts
IA-2 (PTPRN)	IA-2 752–775 (overlapping B/T epitopes)	Variable	Late-appearing autoantibodies; rapid progression marker	[20,21]; epitope overlap confirmed by multiple methods
ZnT8 (SLC30A8)	ZnT8 318–331 (VAANIVLTVVAEFLR	Multiple	Polymorphism-driven immunogenicity; adds diagnostic value	[22]; discovery robustly validated in large international cohorts

In addition to these four dominant autoantigens (insulin, GAD65, IA-2, ZnT8), autoreactive T and B cells act on a wider range of islet antigens, including chromogranin A, IGRP, and others, expanding the possibilities of autoimmune therapy [11]. This multiplicity of antigens creates difficulties for antigen-specific therapy directed at a single antigen, since targeting one of them may not completely suppress responses to various epitopes that cause beta-cell destruction [11].

Traditionally, research efforts have predominantly focused on the classical autoantigens identified in T1DM. However, studies increasingly indicate that the immune response in T1DM targets a broader spectrum of antigens, including neoantigens generated through post-translational modifications (Table 2). Hybrid insulin peptides (HIPs) are formed by peptide-bond fusion of insulin fragments (typically C-peptide or B-chain) with peptides from other secretory granule proteins (e.g., IAPP, chromogranin A, WE14). Because these fusions occur only in β-cells and not in the thymus, central tolerance is absent. A well-characterized example is 2.5HIP (insulin B:22–31 fused to WE14: 2–11). Delong et al. first demonstrated that pathogenic CD4^+^T cells from NOD mice and residual islets of human T1DM organ donors recognize HIPs [16]. Baker et al. and Hohenstein et al. subsequently identified HIP-reactive CD4^+^ T cells in peripheral blood and inflamed islets of recent-onset and at-risk individuals, with reactivity detectable years before clinical onset [23,24]. These studies, though performed on limited human samples, have been independently confirmed in multiple laboratories and across species [25], establishing HIPs as one of the most promising pathogenic neoantigens.

Defective ribosomal insulin products (DRiPs) arise from translational errors or frameshifts during insulin synthesis under metabolic stress. Kracht et al. identified autoreactive CD8^+^ T cells specific for a defective insulin variant in T1DM patients [26]. Although the exact peptide sequences remain proprietary in some follow-up studies, the concept is mechanistically compelling and supported by parallel observations in other autoimmune diseases.

Post-translational modifications (deamidation, citrullination, oxidation) create additional neoepitopes. The best-documented example is deamidated GAD65 274–286 and multiple deamidated isoforms of InsB:9–23, which bind with far higher affinity to DQ8 than the native sequence [16]. The restricted TCR repertoire recognizing these modified and hybrid epitopes, combined with their absence from thymic selection, makes them exceptionally attractive targets for next-generation antigen-specific immunotherapies.

A subsequent stage in TCR therapy development involves identifying autoantigen-specific TCRs via sequencing methodologies. The candidate TCRs undergo rigorous validation for high expression, specificity, and reactivity. The leading TCR is then selected, followed by the production of viral vectors for transduction into T cells. Several research groups have reported detailed protocols for the identification of pathogenic TCRs and employing them to develop TCR-based therapy for various autoimmune diseases, including T1DM [27,28,29,30,31]. TCR identification is typically achieved by analyzing T cell clones isolated from pancreatic infiltrates or peripheral blood of T1DM patients. A number of TCR clonotypes specific for INS, GAD65, IA-2, and the catalytic subunit 2 of glucose-6-phosphatase (G6PC2) have already been isolated from autoreactive T cells via sequencing [28,29,32,33].

The detection and characterization of pathogenic TCRs directed against key autoantigens (e.g., INS, GAD65, IA-2) and particularly against post-translationally modified forms such as HIP, represent a critical step in elucidating T1DM pathogenesis. The identification of immunodominant epitopes and the comprehensive analysis of the autoreactive T cell TCR repertoire have enabled the pinpointing of promising targets for the development of antigen-specific immunotherapy approaches.

## 3. Modulating Autoreactive T Cell Clones and Their Role in T1DM Pathogenesis

T1DM is characterized by an elevated frequency of islet-specific autoreactive CD8^+^ T cells, often occurring in conjunction with a diminished immune regulatory capacity [6]. The critical role of the T cell-mediated immune response in T1DM pathophysiology is corroborated by several key observations, such as: the development of the disease following allogeneic bone marrow transplantation without T cell depletion [34]; the development of T1DM in individuals with B cells and antibody deficiencies [35]; and hereditary genetic defects in T cell functions that are associated with diabetic manifestation [36].

The vast majority of studies investigating peripheral autoimmunity in T1DM patients reveal significant phenotypic overlap with that observed in the general population. Concurrently, the prevalence of circulating islet-autoreactive cells is frequently exceedingly low. This poses substantial obstacles to establishing a definitive correlation between the peripheral pool of autoreactive immune cells and the localized pathological processes within the islets of Langerhans. A significant breakthrough involved the direct isolation of T cells specific for beta-cell antigens from the islets of T1DM donors [37,38,39]. The histopathological hallmark of these immune processes is insulitis, characterized by immune cell infiltration into the pancreatic islets [40]. Within the insulitic infiltrate, CD8^+^T cells represent the dominant cellular component, while CD4^+^T cells are present in smaller numbers. Studies have identified specific histological patterns of insulitis that correlate with the severity of beta-cell destruction and the age of disease onset [41]. Importantly, in contrast to experimental animal models, which typically display pronounced and widespread infiltration, insulitis in humans is observed less frequently and is characterized by notable heterogeneity in its histological presentation [42].

Insulin-specific CD8^+^ T cells play a pivotal role in the pathogenesis of diabetes in both murine models [43] and humans [44]. Consequently, the development of therapeutic strategies targeting the depletion of cytotoxic CD8^+^T cells is pathogenetically justified and holds significant promise. A study by McKinney et al. outlines a strategy for inducing CD8^+^T cell exhaustion in the context of infectious and autoimmune diseases [45]. Notably, in contrast to viral infections, where CD8^+^T cell depletion is often associated with an unfavorable prognosis, this approach correlates with a favorable long-term outcome in autoimmune and inflammatory conditions. CD8^+^T cells undergoing exhaustion exhibit increased expression of co-inhibitory receptors (e.g., PD-1) and suppression of formative memory markers (e.g., IL7R). This state of exhaustion is induced by the persistent presentation of antigen in the absence of adequate co-stimulation. To recapitulate in vitro the signatures associated with disease outcome, purified human CD8^+^T cells were stimulated with magnetic particles conjugated to antibodies targeting co-stimulatory molecules, with subsequent assessment of PD-1 and IL7R expression. Results demonstrated that transient (36 h) TCR stimulation enabled cells to restore IL7R expression post-division, whereas prolonged (6-day) stimulation impeded this restoration. Given that blockade of co-inhibitory signals (e.g., PD-1) can reverse established functional exhaustion, the amplification of these inhibitory signals represents a potential therapeutic strategy in autoimmune diseases for inducing a state of exhaustion in the setting of a hyperimmune response.

In a related study investigating the treatment of ankylosing spondylitis, a research group identified CD8^+^T cells expressing TRBV9 as being associated with the pathogenesis of ankylosing spondylitis, psoriatic arthritis, and acute anterior uveitis. Related epitopes presented by HLA-B*27 were also identified [46]. Following successful validation in non-human primate models, the authors report the selective removal of TRBV9^+^T cells in individuals with ankylosing spondylitis. This intervention led to disease remission, and treatment with tumor necrosis factor inhibitors was successfully discontinued after 5 years of sustained therapy. Such targeted elimination of the disease’s root cause, achieved without systemic immunosuppression, could represent a new generation of safe and effective therapeutic modalities for autoimmune diseases.

## 4. TCR-Based Tolerance Induction

Central to the rationale for TCR-Treg therapies in T1DM is the established defect in Treg function and frequency. In T1DM patients, Tregs exhibit impaired suppressive capacity, reduced *FOXP3* expression, and diminished IL-10/TGF-β production, failing to counterbalance autoreactive effectors [47]. This immunological dysfunction underscores the need for TCR-Treg to restore tolerance.

Contemporary approaches to the management of T1DM increasingly incorporate innovative immunotherapeutic strategies, with Tregs playing a central role. Pioneering clinical applications of autologous Tregs in humans were first reported by Trzonkowski and colleagues, who initially used ex vivo-expanded Tregs to successfully treat graft-versus-host disease [48] and subsequently demonstrated their safety and ability to preserve beta-cell function in children with recent-onset T1DM [49]. Building on these landmark studies, one of the most promising current directions is the development of TCR-Tregs. The principles, advantages, and limitations of TCR-Treg-based and alternative approaches are summarized in Table 3. This advanced technology involves transferring TCR genes from autoreactive Teff into Tregs, thereby generating a highly antigen-specific population capable of recognizing relevant autoantigenic epitopes. TCR gene delivery is achieved using lentiviral or retroviral vectors for stable transgene expression [50] or the CRISPR-Cas9 system for precise integration into predefined genomic loci [51]. The era of TCR-based therapy began with cancer research. Therefore, we have the most data on methods for delivering TCRs to T cells, which are also applicable to Tregs.

Lentiviral vectors remain the most widely used platform for ex vivo T cell engineering due to their high transduction efficiency and durable transgene expression. Recent clinical data from MAGE-A4–directed TCR-T cell programs have reinforced their utility: a multicenter phase 1 trial of afamitresgene autoleucel demonstrated acceptable safety and encouraging antitumor activity in patients with MAGE-A4-positive solid tumors, including synovial sarcoma [52,53]. Continued improvements in vector architecture—including self-inactivating (SIN) designs—and large-scale manufacturing have strengthened the safety profile of lentiviral products, though long-term insertion-site monitoring remains recommended [54,55]. Gamma-retroviral vectors, the earliest vectors used for T cell gene transfer, remain clinically relevant in defined settings because of their mature production processes and documented capacity for long-term TCR expression. Recent reviews of TCR-T and related adoptive therapies highlight durable persistence of γ-retrovirally engineered T cell clones in multiple programs, while again noting the need for insertion-site surveillance [56,57].

As a non-viral alternative, the Sleeping Beauty (SB) transposon system has progressed into a feasible platform for clinical-scale TCR-T and CAR-T manufacturing. Several translational and early clinical studies have shown that SB-engineered products can be produced at reduced cost and with shorter manufacturing times relative to viral methods, supporting the system’s growing relevance for off-the-shelf applications [58,59].

The most transformative advances in TCR engineering have come from CRISPR-Cas9 genome editing. Modern CRISPR platforms enable simultaneous knockout of endogenous genes and targeted knock-in of therapeutic TCRs at the TRAC locus. Recent clinical and translational work shows that CRISPR-engineered TCR-T cells exhibit improved persistence, reduced mispairing, and enhanced antitumor activity [60,61]. TCR mispairing continues to be an important focus of engineering innovation. Beyond CRISPR-mediated endogenous TCR disruption, recent strategies include the use of murine-derived constant domains, engineered cysteine bonds, and other sequence-level modifications. Comparative analyses suggest that combinations of targeted TRAC integration and structural engineering approaches most effectively minimize mispairing and improve functional avidity [60,62].

Preclinical studies in NOD murine models have demonstrated the robust efficacy of TCR-Tregs targeting proinsulin, pancreatic islet cells, and GAD65 epitopes [28,29,63]. Utilizing TCRs derived from CD4^+^T cells, antigen-specific TCR-Tregs were generated through viral transduction, which effectively suppressed the proliferation of Teffs and their cytokine production. In a study by Yang et al. [63], adoptively transferred murine-derived engineered Tregs specific for pancreatic islets exhibited targeted localization to the pancreas and abrogated diabetes development induced by islet-specific Teffs. These compelling findings underscore the therapeutic potential of antigen-specific TCR-Tregs as a targeted intervention for T1DM prevention.

Of particular translational interest is the strategic targeting of TCR-Tregs towards epitopes derived from post-translationally modified autoantigens, such as deamidated GAD65 and hybrid insulin peptides, which exhibit heightened immunogenicity [64]. Wiles et al. [65] demonstrated that the BDC-6.9 T cell clone (a diabetogenic CD4^+^T cell line from NOD mice) exhibits reactivity against pancreatic islet cells. Hybrid insulin peptide 6.9 (6.9HIP), synthesized from insulin C-peptide and islet amyloid polypeptide (IAPP), functions as a cognate antigen for the BDC-6.9 T cell clone. Mass spectrometric analysis confirmed the endogenous presence of 6.9HIP within pancreatic beta cells. An MHC class II tetramer reagent (6.9HIP-tet) revealed a marked enrichment of T cells specific for 6.9HIP within the pancreatic environment of diabetic NOD mice. Further investigation of HIP and their cognate T cell interactions may facilitate the rational design of more targeted and efficacious therapeutic interventions for T1DM.

The primary advantages of employing post-translationally modified antigens stem from their heightened specificity (as modified epitopes are rarely encountered in healthy tissues) [66] and amplified immunogenicity [38]. However, several challenges persist, including the insufficient identification of all clinically relevant modified antigens and the potential risk of inducing novel autoreactive clones when utilizing highly immunogenic epitopes.

In the context of TCR-Treg development, careful consideration must be given to the source and specificity of the TCR. Utilizing TCRs derived from pathogenic T cells that infiltrate the islets of Langerhans may prove beneficial, as these receptors are specific for antigens that are appropriately expressed and localized to elicit an immune response. Studies predominantly employ TCRs obtained from islet-reactive CD4^+^T cells [28,29,63], whereas TCRs derived from islet Tregs have been investigated to a minimal extent [67]. Comprehensive comparative analyses evaluating the efficacy of TCRs derived from Tregs versus Teffs remain limited. Furthermore, the effectiveness of targeting specific antigens, such as hybrid peptides, remains unclear due to a lack of in vivo comparisons of TCR antigen specificity.

A key clinical challenge for Treg therapies in human autoimmunity is in vivo viability and persistence. Infused Tregs often face inflammatory environments that destabilize *FOXP3* expression, leading to conversion to effector phenotypes or apoptosis, necessitating strategies like chemical-inducible signaling or multi-factorial engineering for enhanced durability [68]. A promising approach to address this issue is to convert Teff into antigen-specific Tregs with a stable phenotype through dual transduction. For instance, Wright et al. transferred *FOXP3* and TCR genes into CD4^+^Teffs to generate antigen-specific TCR-Tregs for adoptive T cell therapy in arthritis models [69]. They employed the OTII-TCR, which targets SERPIN (serine protease inhibitors) presented by MHC class II molecules. This strategy resulted in a localized reduction in inflammatory Th17 cells and a significant mitigation of bone destruction [69].

In a related study, Uenishi et al. [32] reported a strategy involving the reprogramming of conventional CD4^+^T cells to co-express *FOXP3*, an engineered IL-2 receptor responsive to rapamycin, alongside a TCR specific for a pancreatic islet autoantigen. The resultant cellular product, designated GNTI-122 TCR-Tregs, exhibited stable *FOXP3* expression, demonstrated targeted migration to the pancreas and draining lymph nodes, and incorporated a chemically inducible signaling complex (CISC). Rapamycin-mediated activation of the CISC prompted dose-dependent phosphorylation of STAT5 and, in concert with TCR engagement, facilitated cellular proliferation. Upon encountering the cognate antigen, these engineered Tregs elicited both direct and indirect suppression of polyclonal, islet-specific Teffs in individuals with T1DM.

The determination of optimal TCR affinity for eliciting therapeutic efficacy in autoimmune disorders remains a subject of intensive investigation. While the use of high-affinity TCRs might appear intuitively advantageous, emerging evidence suggests a more complex scenario [70,71]. Sprouse et al. [70] demonstrated that both high- and low-affinity Tregs were capable of migrating to the pancreas and executing their suppressive functions. High-affinity Tregs mediated their effects via IL-10, TIGIT, GITR, and CTLA4, whereas low-affinity Tregs exhibited augmented expression of Areg and Ebi3 genes, underscoring the heterogeneity in their functional profiles and emphasizing the potential contribution of both high- and low-affinity TCRs [70]. Furthermore, Wyss et al. [71] supported the concept that the affinity of the TCR for autoantigens shapes the generation of Tregs exhibiting distinct functional characteristics. Consequently, future research efforts are warranted to delineate the precise TCR affinity profile for restoring immune tolerance in autoimmune diseases, possibly through the application of TCRs with varied affinities.

An alternative and innovative approach involves engineering Treg specificity towards a TCR that exhibits cross-reactivity with multiple autoantigens. In a study by Malviya et al. [27], myelin oligodendrocyte glycoprotein (MOG) and neurofilament mediator (NF-M) were utilized as target antigens, demonstrating that the use of a cross-reactive TCR targeting multiple autoantigens was more effective than strategies employing monospecific TCR-Tregs. Engineered the MOG/NF-M cross-reactive TCR-Tregs displayed enhanced immunoregulatory activity compared to the MOG-specific TCR-Tregs in a murine model of experimental autoimmune encephalomyelitis (EAE) induced by MOG. Notably, the MOG/NF-M cross-reactive TCR-Tregs also improved the disease course in EAE induced by an unrelated central nervous system (CNS) autoantigen, proteolipid protein (PLP).

New technologies are complementing TCR-Treg, such as self-antigen-loaded HLA class II nanoparticle (pMHCII-NP) platforms that engage and expand type 1 cytotoxic regulatory cells (Tr1) in type 1 diabetes, facilitating targeted suppression [72]. Similarly, chimeric antigen receptor (CAR)-Treg cells expressing specific HLA-A2 receptors (A2-CAR-Treg) have shown promising results in a preclinical model, inducing linked suppression and durable tolerance to a specific antigen [73]. These innovations expand the capabilities of Tregs beyond TCR engineering.

The successful translation of TCR-Treg therapy for T1DM is contingent upon several critical factors: the optimization of genetic modification strategies, the enhancement of Treg in vivo stability, and the development of combinatorial therapeutic approaches, encompassing the targeting of multiple modified autoantigens. Despite the inherent technological complexities, this innovative approach constitutes a potent tool for re-establishing immune tolerance in individuals affected by T1DM, offering the dual benefits of high specificity and minimized systemic immunosuppression. Optimal TCR-Treg therapy in T1DM involves the integration of high-affinity, multiantigen-specific T cells (targeting classical and neoantigens, such as HIP) into stable *FOXP3*^+^CD4^+^cells in combination with cell survival enhancers (e.g., IL-2 signaling domains). This model will enable suppression of the autoimmune process while minimizing off-target effects and simultaneously maximizing tolerance induction. Efficacy would be greatest in Stage 1 (presymptomatic, autoantibody-positive) for prevention via early tolerance restoration, moderately effective in Stage 2 (dysglycemia) to delay onset, and challenging but possible in Stage 3 (clinical T1DM) if residual beta cells persist, requiring combination with beta-cell protectants [72].

Considering the therapeutic potential of TCR-Treg strategies for T1DM, it is imperative to also explore other promising avenues in the broader field of immunotherapy for autoimmune diseases, with CD8^+^Tregs representing a particularly compelling target for future investigation.

## 5. CD8^+^Tregs

CD8^+^Tregs represent a potential therapeutic tool for autoimmune diseases, including T1DM. In contrast to classical CD4^+^Tregs, CD8^+^Tregs exhibit a narrower antigen specificity and possess the capacity to effectively inhibit the proliferation and function of autoreactive T cells through mechanisms such as the secretion of immunomodulatory cytokines (e.g., IL-10 and TGF-β) and direct cytotoxic effects [74,75]. The model proposed by Jiang et al. [74] posits that antigen-specific CD8^+^Tregs can selectively suppress the activity of antigen-activated T cells expressing TCRs with specific affinities. Consequently, their findings suggest that CD8^+^Tregs can modulate the peripheral TCR repertoire during immune responses to both self and foreign antigens.

While CD8^+^Tregs are frequently described as a homogeneous population, several researchers have proposed their classification into distinct subpopulations. These include CD8^+^*FOXP3*^+^T cells, CD8^+^CD122^+^T cells, CD8^+^CD28^low/−^T cells, CD8^+^CD45RC^low^T cells, T cells expressing the CD8αα homodimer, and CD8^+^T cells with restricted reactivity to Qa-1 [76]. It is noteworthy that co-expressed markers on some putative CD8^+^Treg subsets indicate potential functional overlap [76].

CD8^+^Treg development can occur within peripheral lymphoid organs during an immune response. Continuous antigenic stimulation, a key determinant for the generation of adaptive Tregs, has been shown to induce the differentiation of highly suppressive adaptive CD8^+^CD25^+^*FOXP3*^+^Tregs [77]. The resulting CD8^+^CD25^+^*FOXP3*^+^Tregs effectively inhibit the proliferation and cytokine production of CD4^+^ and CD8^+^T cells, without impairing perforin and granzyme expression in CD8^+^Teffs. A crucial mechanism for peripheral induction involves interactions with other immune cells. For instance, in vitro studies have demonstrated that co-cultivation of CD8^+^CD28^−^T cells with monocytes leads to the formation of suppressor cells whose function is IL-10 dependent [78]. Similarly, stimulated CD3^+^ bone marrow stromal cells have been shown to induce the differentiation of human CD8^+^CD28^+^*FOXP3*^+^Tregs [79].

The capacity of CD8^+^Tregs to suppress inflammatory responses is partly attributable to their influence on autoreactive cells, as well as effector CD4^+^T cells. Several studies have demonstrated that CD8^+^Tregs inhibit activated antigen-specific CD4^+^T cells expressing Qa-1 [74]. Given that Qa-1 exhibits binding affinity for autopeptides that activate natural killer receptors on CD8^+^T cells, it is plausible that Qa-1 plays a critical role in maintaining CD8^+^Treg homeostasis [74].

Beyond modulating T cell-mediated immunity, CD8^+^Tregs can also exert influence on the B cell compartment. This effect is associated with Qa-1 expression, as CD8^+^Tregs inhibit T-follicular helper cells (Tfh), characterized by active Qa-1 expression, thereby leading to diminished germinal center (GC) formation and impaired antibody maturation [80]. Studies in ApoE mice crossed with Qa-1 or Qa-1 D227K mice have revealed that CD8^+^Treg dysfunction accelerates the progression of atherosclerosis, potentially driven by enhanced Tfh and GC activity [81].

A recent study elucidating the mechanisms by which CD8^+^Tregs mediate immunosuppression in diabetes suggests a role for trehalose, a nematode-derived substance that, through its influence on the microbiota, promotes an increased abundance of *Ruminococcus* spp [82]. This trehalose-driven expansion of *Ruminococcus* has been shown in preclinical mouse models to correlate with enhanced CD8^+^Treg activity and reduced diabetogenic inflammation [82]. Beyond *Ruminococcus*, several microbiota-derived factors have been proposed to influence regulatory T cell responses. For example, polysaccharide A (PSA) from Bacteroides fragilis induces IL-10–secreting regulatory T cells, including CD8^+^ subsets, in preclinical models [83,84], while short-chain fatty acids (SCFAs), produced primarily by fermenting Clostridium clusters, support regulatory T cell programs through epigenetic and metabolic mechanisms [85]. Although most mechanistic data concern CD4^+^Tregs, these pathways provide plausible biological routes through which additional taxa might modulate CD8^+^Treg differentiation.

Despite the promise of CD8^+^Tregs as novel candidates for the development of TCR-Treg therapy, several critical questions remain unanswered, and conflicting data have emerged across various studies. Notably, the extent of overlap between individual CD8^+^Treg populations is not yet fully understood. There is a relative paucity of studies directly comparing putative CD8^+^Treg subpopulations, particularly in vivo. Furthermore, data about the stability of the regulatory phenotype of these cells under inflammatory conditions are limited. Analogous to classical Tregs and CD8^+^ counterparts, the effectiveness and safety of this therapeutic approach depend largely on the interaction of modified cells with various components of the immune system. Therefore, a more comprehensive and detailed investigation into the impact of this therapeutic strategy on other immune cell types is necessary to fully appreciate the potential of TCR-Treg therapy.

## 6. Impact of TCR-Treg Therapy on Other Cells of the Immune System

**Figure 2:**
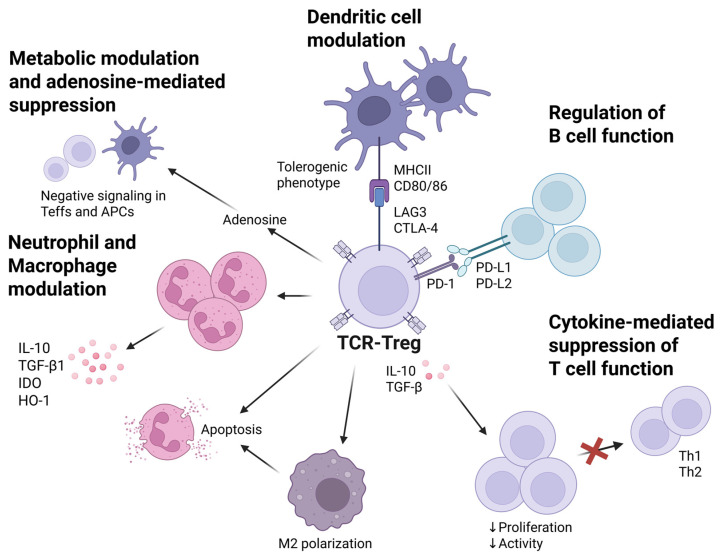
Impact of TCR-Treg Therapy on the Immune System: Cellular and Molecular Mechanisms. Tregs exert immunomodulatory effects on diverse immune cell populations, including those implicated in disease pathogenesis. Neutrophil and macrophage modulation: Tregs induce neutrophil apoptosis, promote the formation of secondary immunosuppressive neutrophils that produce IL-10, TGF-β1, indoleamine 2,3-dioxygenase (IDO), and heme oxygenase 1 (HO-1), and stimulate the phagocytosis of neutrophils by macrophages, thereby facilitating macrophage polarization towards an anti-inflammatory M2 phenotype. Dendritic cell modulation: Tregs engage with DCs, disrupting their maturation and antigen presentation processes through the interaction of CTLA-4 with CD80/CD86 molecules and by binding LAG3 to MHC class II molecules on DCs, which promotes the generation of DCs with a tolerogenic phenotype. Cytokine-mediated suppression of T cell function: IL-10 directly inhibits T cell proliferation, suppresses antigen presentation, and diminishes DC activity, whereas TGF-β inhibits T cell proliferation and the differentiation of Th1 and Th2 cells. Metabolic modulation and adenosine-mediated suppression: Tregs orchestrate the suppression of T cell activity by modulating metabolic processes, including the production of adenosine, resulting in negative signaling within effector T cells and antigen-presenting cells. Regulation of B cell function: Tregs regulate autoimmune B cell activity via binding to PD-1 on autoreactive B cells through interactions with PD-L1 and PD-L2 expressed on Tregs. Furthermore, Tregs may utilize granzymes A and B, as well as perforin, to indirectly suppress the activity of effector B and T cells.

Immunoregulatory strategies based on the adoptive transfer of TCR-Tregs have demonstrated considerable therapeutic promise for the treatment of autoimmune disorders and the prevention of allograft rejection. To understand the mechanisms underlying TCR-Treg function, it is imperative to consider their multifaceted impact on other cellular constituents of the immune system (Figure 2). While the core immunoregulatory functions of TCR-Tregs largely mirror those of non-specific Tregs, antigen specificity engenders unique characteristics. The predominant mechanism of action involves the suppression of Teff activation and proliferation, a process requiring both direct cell–cell contact and the secretion of soluble mediators, in particular TGF-β, IL-10, and IL-35 [86,87,88]. Notably, the immunosuppressive potency of TCR-Tregs is significantly amplified upon prior activation via the TCR, highlighting the critical importance of antigen-specific targeting for achieving optimal therapeutic efficacy [89,90,91]. At the molecular level, this suppression is mediated by several mechanisms, including competition for IL-2 via the high-affinity CD25 receptor, induction of tryptophan catabolism through indoleamine 2,3-dioxygenase (IDO) [92,93], and the transmission of inhibitory signals through CTLA-4–CD80/86 and PD-1–PD-L1 interactions [94,95]. Adenosine generation from ATP, catalyzed by CD39 and CD73 expressed on activated Tregs, represents another key mechanism, leading to suppression of T cell function by inducing negative signaling in Teffs and antigen-presenting cells (APCs) [96]. Similarly to their conventional counterparts, TCR-Tregs are capable of modulating dendritic cell (DC) function. Tregs induce a state of immune tolerance in DCs through CTLA-4 interaction with CD80/CD86 molecules, inhibiting DC maturation and reducing the production of proinflammatory cytokines, such as IL-12 and TNF-α [97]. Furthermore, TCR-activated Tregs promote the expression of the immunoregulatory molecules PD-L1 and IDO in DCs, which establishes an additional suppressive axis for Teffs [98]. These effects can be mediated through both direct cell contact and the release of IL-10 and TGF-β.

Given their antigen specificity, TCR-Tregs can be selectively deployed for the immune regulation of diseases, potentially enabling the administration of lower cell doses and reducing the risk of systemic immunosuppression [99]. It has been demonstrated that antigen-specific Tregs are capable of removing MHC class II peptide complexes from the surface of DCs, thus precluding the activation of naïve Teffs by DCs [100,101]. Emerging evidence suggests that Tregs also attenuate the capacity of APCs to present autoantigens to pathogenic T cells, which facilitates the establishment of a tolerogenic phenotype in DCs, promoting the expression of IL-10 and TGF-β. For instance, Eggenhuizen et al. [102] demonstrated that co-culturing Tregs with GBM-TCR enhances the production of IL-10 and TGF-β by DCs, thereby inducing their tolerogenic phenotype.

Macrophages also represent an important target for Treg-mediated regulation. Tregs have been shown to reprogram macrophages from the proinflammatory M1 phenotype to the anti-inflammatory M2 phenotype, as well as attenuate macrophage activation [103]. Moreover, Tregs can locally contribute to reduced macrophage infiltration. In a study by Dantas et al. [104], Tregs diminished the number of macrophages in affected skin, which resulted in a slowing of disease progression in a murine model of psoriasis. This immunomodulatory reprogramming has the potential to establish a sustained immunosuppressive microenvironment, a feature highly desirable for the effective management of chronic autoimmune diseases.

Neutrophils are also subject to Treg influence. Umeshappa et al. [105] have shown that autoantigen-specific T-regs utilize immunoregulatory cytokines to coordinate neutrophil recruitment to the liver and to program their transcriptome towards a regulatory phenotype. Recent studies have further revealed that antigen-specific Tregs can inhibit the formation of neutrophil extracellular traps (NETosis), a process critically implicated in the pathogenesis of systemic lupus erythematosus and vasculitis [106]. This effect may be attributed to the ability of Tregs to diminish the production of reactive oxygen species in neutrophils.

The influence of TCR-Tregs on B cells constitutes another important facet of their immunoregulatory activity. Direct inhibition of B cells by Tregs is contingent upon two key conditions: the expression of PD-1 on autoreactive B cells and the expression of PD-1 ligands (PD-L1 and PD-L2) on Tregs. Furthermore, Tregs utilize granzyme B and perforin (which facilitate pore formation in the plasma membrane) to reduce autoantibody production by B cells, thereby mediating their immunosuppressive effects [107].

There is interesting evidence that mast cells may play an important role in Treg-dependent peripheral tolerance. In a study by Li-Fan Lu et al. [108], it was revealed that IL-9 is instrumental in the process by which activated Tregs recruit and activate mast cells, contributing to regional immune suppression. Neutralization of IL-9 led to a significant acceleration of allograft rejection in tolerant mice, underscoring the importance of this mechanism. Immunohistochemical analysis confirmed the presence of a link between Tregs, IL-9, and mast cells in tolerant allografts.

A paramount advantage of TCR-Treg therapy lies in its capacity to establish long-term immunological memory. In contrast to conventional pharmacological immunosuppressants, transplanted antigen-specific Tregs are able to persist within the recipient organism for extended periods and respond to disease relapses [68].

In summary, the immunosuppressive properties of TCR-Tregs are realized through a multilevel network of mechanisms, encompassing the secretion of inhibitory cytokines (e.g., IL-10, TGF-β, IL-35), metabolic modulation (adenosine generation via CD39/CD73), cytolysis (mediated by granzymes and perforin), modulation of APC function, and direct suppression of B cells (Figure 2). Despite substantial progress, further research is warranted to address key outstanding questions about optimal protocols for TCR-Treg generation, the durability of their regulatory phenotype in vivo, and potential therapeutic risks. Further investigations focused on elucidating tissue-specific interactions and enhancing the specificity of TCR-Tregs will unlock new avenues for the development of personalized immunotherapeutic strategies for the treatment of autoimmune and inflammatory disorders.

## 7. Future Perspectives

Antigen-specific TCR-mediated therapy for T1DM represents a promising alternative to traditional treatment modalities and lies at the forefront of immunological research. Progress in this area encompasses the development of strategies aimed at cytotoxic T cell depletion and the application of TCR-engineered Treg. Despite the advances achieved, further development necessitates addressing several key challenges and capitalizing on emerging opportunities. Although much of the foundational data on TCR-based therapies has been obtained from studies in animal models of NOD, which mimic key aspects of T1D pathogenesis, they are limited in fully capturing the heterogeneity and progression of the disease in humans. Therefore, clinical trials in patients are essential to obtain a complete picture of the efficacy and safety of TCR-based therapies.

Contemporary approaches to studying autoreactive T cells in autoimmune diseases increasingly leverage peptide-MHC multimer technology and comprehensive TCR repertoire analysis. Peptide-MHC multimers, comprising complexes of MHC molecules loaded with cognate peptides and stabilized within a multimeric structure incorporating a fluorescent label, afford high-avidity binding to TCRs, thereby facilitating the selective identification of even rare populations of antigen-specific T cells [109]. The experimental workflow encompasses the procurement of biological specimens, incubation with peptide-MHC multimers, and flow cytometric analysis to identify T cells exhibiting multimer binding, with optional cell sorting to enable downstream analyses. The utilization of multimers confers enhanced specificity, permits direct ex vivo detection in the absence of in vitro stimulation, and provides the capacity for both quantitative assessment and cell sorting of antigen-specific T cells. Subsequent to the isolation of antigen-specific T cells, TCR sequencing is performed to delineate the sequences of the α and β chains responsible for recognizing specific autoantigens [110]. Comprehensive analysis of the TCR repertoire enables the identification of TCR clonotypes, assessment of repertoire diversity, detection of shared TCRs among patients exhibiting a common disease phenotype, and interrogation of the relationship between the TCR repertoire and clinical manifestations. The integration of peptide-MHC multimer technology with comprehensive TCR repertoire analysis unlocks new avenues for studying autoreactive T cells, enabling their ex vivo identification and characterization while preserving their in vivo phenotype [109]. These strategies have been successfully implemented in the development of anti-cancer therapeutics [111], and their application in the domain of autoimmune diseases holds considerable promise.

To optimize tolerance induction strategies, it is imperative to enhance the phenotypic stability of TCR-Tregs and to further explore the therapeutic potential of CD8^+^Tregs, particularly those that are TCR-engineered, given their capacity to infiltrate inflamed tissues and to exert local suppression of pathogenic immune responses. A synergistic approach that combines multiple antigen-specific strategies, such as TCR-Tregs targeting distinct autoantigens, or the integration of TCR-Treg therapy with other immunomodulatory agents, may ultimately prove most effective. Furthermore, sustained research efforts are warranted not only to further elucidate the intricate mechanisms of action of TCR-Tregs but also to comprehensively characterize their effects on other cellular components of the immune system and to assess their long-term therapeutic potential.

In our opinion, TCR-based antigen-specific therapies hold transformative potential for T1DM, shifting from symptomatic insulin replacement to curative tolerance induction. While challenges like TCR affinity optimization and Treg stability persist, the integration of human-derived neoantigen data (e.g., HIP) with advanced tools such as peptide-MHC multimers could accelerate translation. We anticipate first-in-human trials of TCR-Tregs within 5-10 years, potentially achieving long-term remission without systemic risks, especially if combined with beta-cell preservation strategies. However, rigorous safety monitoring and multi-antigen targeting will be essential to overcome current limitations.

## 8. Literature Search Strategy

This review was conducted through a systematic search of peer-reviewed literature in PubMed, Scopus, and Web of Science databases. Search terms included combinations of: “type 1 diabetes” OR “T1D” OR “autoimmune diabetes”, “TCR” OR “T cell receptor”, “antigen-specific”, “TCR-Treg” OR “regulatory T cell”, “tolerance induction”, “autoreactive T cell”, “insulin”, “GAD65”, “IA-2”, “ZnT8”, “hybrid insulin peptide” OR “HIP”, “TCR editing”. Inclusion criteria: (1) studies in English; (2) original research or reviews on TCR-based or antigen-specific immunotherapy in T1DM models or humans; (3) focus on T cell modulation, Treg engineering, or autoantigen identification. Exclusion criteria: non-T1DM autoimmune models without T1DM relevance, non-TCR-based therapies, and studies lacking mechanistic insight. 112 references were selected based on relevance, impact, and recency, with priority given to human data where available. The search was supplemented by cross-referencing from key reviews and manual curation of seminal works.

## 9. Limitations of the Review

Although this review aims to provide an up-to-date summary of TCR-based antigen-specific strategies for type 1 diabetes, several limitations should be acknowledged. First, most mechanistic data on engineered T cells and regulatory T cell therapies are derived from preclinical studies, which may not fully reflect human disease heterogeneity. Second, the rapidly evolving nature of TCR engineering, genome editing, and neoantigen discovery means that emerging findings may not be fully captured at the time of publication. Finally, as the antigenic landscape of type 1 diabetes and the clinical translation of TCR-based cell therapies continue to develop, some interpretations presented here should be viewed as provisional. Further research, including future systematic reviews and meta-analyses, will help clarify these aspects and strengthen the evidence base needed to refine and validate the therapeutic concepts discussed.

## Figures and Tables

**Table 2:** Neoantigens in T1DM.

Neoantigen Type/Approach	Specific Example	Formation Mechanism/Concept	Key Observations and Limitations	References
Hybrid Insulin Peptides (HIP)	2.5HIP; 6.9HIP (Ins C-peptide + IAPP)	Peptide-bond fusion in β-cell granules	Enriched in NOD islets; T cell reactivity in human blood and islets precedes clinical onset	[16,23,24,25]
Defective Ribosomal Products (DRiPs)	Defective insulin-derived peptides	Translational errors under stress	Autoreactive CD8^+^ T cells in patients; limited human data so far	[26]
Post-translational Modifications	Deamidated InsB:9–23 (Q→E at multiple positions); deamidated GAD65	Tissue transglutaminase, inflammation	Dramatically increased binding/immunogenicity; reproducible across cohorts	[16]

**Table 3:** Principles, advantages, and limitations of TCR-Treg-based and alternative approaches.

Therapy Type/Approach	Principle of Action	Advantages	Limitations/Considerations
TCR-Tregs targeting classical autoantigens	TCRs specific for INS, GAD65, IA-2, ZnT8 epitopes.	Well-studied epitopes; known HLA restrictions.	Single-epitope targeting may be insufficient.
TCR-Tregs targeting neoantigens (HIPs, DRiPs, deamidated epitopes)	TCRs recognizing modified/hybrid autoantigens.	High specificity; potentially stronger suppression.	Incomplete neoantigen mapping; immunogenicity risks.
Dual transduction: *FOXP3* + TCR	Reprogramming Teff into stable Tregs by *FOXP3* + antigen-specific TCR.	Enhanced stability; resistance to inflammation.	Complex engineering; may need IL-2 support.
Cross-reactive/multi-specific TCR-Tregs	TCRs recognizing multiple related autoantigens.	Broader suppression; suitable for multi-antigen diseases.	Off-target risks; complex validation.
CAR-Tregs	CAR-mediated antigen recognition independent of HLA.	HLA-independent; tunable signaling.	Non-physiologic signaling; limited surface targets.
pMHCII nanoparticles	Activation/expansion of antigen-specific Tr1/Tregs without gene editing.	Non-genetic; safe; personalized.	Lower potency; requires repeat dosing.
CD8^+^ Treg-based therapies	Utilization or engineering of CD8^+^regulatory subsets.	Strong suppression; modulates T and B cells.	Phenotypic instability; limited clinical data.

## Data Availability

No new data were created or analyzed in this study. Data sharing is not applicable to this article.
